# Effects of probiotics on selected anthropometrics and biochemical measures in overweight or obese Saudi subjects: a double-blind, placebo-controlled, randomised clinical trial

**DOI:** 10.1017/S1368980024002003

**Published:** 2024-11-06

**Authors:** Samira M AlMalki, Hanan A Alfawaz, Turki A Binmoammar, Saleh F AlBahlei, Luluah M Al Bakr, Ahmed M Alzahrani, Salem S Alshammari, Syed Danish Hussain, Shaun Sabico, Nasser M Al-Daghri

**Affiliations:** 1 Department of Food Science and Nutrition, College of Food & Agriculture Science, King Saud University, Riyadh, Saudi Arabia; 2 Department of Family and Community Medicine, College of Medicine, King Saud University, Riyadh, Saudi Arabia; 3 Department of Family and Community Medicine, College of Medicine, King Saud University Medical City, King Saud University, Riyadh, Saudi Arabia; 4 Biochemistry Department, College of Science, King Saud University, Riyadh, Saudi Arabia

**Keywords:** Probiotic supplementations, Multi-strain probiotics, Anti-obesity, Weight reduction

## Abstract

**Objective:**

This study aimed to assess the effects of multi-strain probiotics on anthropometric and biochemical measures in Saudi adults with overweight or obesity.

**Design:**

Single-centre, double-blind, placebo-controlled, randomised clinical trial.

**Setting:**

Occupational Health Clinics at King Saud University Medical City, Riyadh, Saudi Arabia.

**Participants:**

Ninety-three Saudi participants with overweight or obesity were randomly assigned to receive twice-daily doses of either placebo (*n* 49) or 30 × 10^9^ CFU/g of HEXBIO® containing three *Lactobacillus* and three *Bifidobacterium* species (*n* 44) in a double-blind manner over a 12-week period, respectively. Both groups adhered to a hypoenergetic diet. Anthropometric measurements, glycaemic indices and lipid profiles were evaluated at baseline and post-intervention.

**Results:**

Following the 12-week intervention, no statistically significant differences were found in all between the probiotic group and placebo group comparisons, except for fat intake, where the group*time interaction showed a significant decrease in favour of the probiotic group (*P* = 0·02). However, significant within-group reductions were observed in the probiotic group: body weight (–0·9 kg, *P* = 0·02), HC (–1·5 cm, *P* = 0·002), energy intake (–387·3 kcal/d, *P* = 0·002), fasting glucose (–0·7, *P* = 0·002) and LDL-cholesterol (–0·7, *P* = 0·02).

**Conclusion:**

Consumption of multi-strain probiotic supplementation over 12 weeks significantly decreased fat intake in Saudi adults with overweight or obesity, with the probiotic group highlighting improved anthropometric and biochemical parameters. Further research is needed to evaluate the long-term clinical significance of this dietary practice and whether it has a meaningful impact on overall health beyond the placebo effect.

Obesity is considered a major public health problem globally. In 2016, almost two billion (39 %) adults 18 years and above were overweight, and 650 (13 %) million had obesity^(1)^. In Saudi Arabia, the percentage of overweight among the Saudi population is 38 %, and the percentage of obesity is 20 %^(2)^. Remarkably, there has been a significant decline of more than 40 % in the occurrence of obesity and overweight among young Saudi individuals from 2012 to 2021. Despite this observable decline, obesity remains prevalent across several demographic factors, including age, sex and geographical distribution, throughout Saudi Arabia^(3)^. Obesity is categorised as a low-grade chronic and systemic inflammatory condition. Extensive research has been conducted to develop treatment strategies and preventive measures for this condition^(4)^. It has been documented that there is an association between obesity and the composition of gut microbiota (GM) in human subjects^(5)^. Many studies reported that the relative proportion of microbiota varies between individuals with obesity and lean people^(6)^. In addition to this, Bombani *et al.* found that the GM population differs considerably depending on the degree of obesity^(7)^.

GM is considered a contributory factor in maintaining energy metabolism and fat storage through many mechanisms^(8)^. Indeed, some probiotics have demonstrated anti-obesity properties and can be used as a complementary technique for obesity management^(9)^. Furthermore, probiotics and synbiotics, whether single strain or multi-strain, may have a positive impact on weight loss and other related anthropometric indices in individuals with overweight or obesity^(10,11)^. Furthermore, some studies observed the effects of probiotics/synbiotics in lowering obesity biomarkers such as oxidative stress^(12)^. However, GM diversity and composition are profoundly influenced by the individual host’s diet, lifestyle and environmental factors^(10,13)^. The ratio of *Firmicutes* to *Bacteroidetes* has also been linked to obesity and sex differences. For example, women exhibited a higher proportion of *Firmicutes* independent of BMI, while males exhibited a greater percentage of *Firmicutes* when their BMI was 33 kg/m^2^ and a lower percentage when their BMI > 33 kg/m^2^. Notable differences between men and women have also been observed in some microbial strains such as the *Bacteroides* genus, with lower counts seen in men with morbid obesity than their leaner counterparts, a finding not seen in women^(14)^.

Dietary supplementation of probiotics for the purpose of altering GM composition is potentially effective in achieving favourable metabolic outcomes. A recent systematic review indicated that certain strains of probiotics, such as *Streptococcus thermophilus*, *Lactobacillus bulgaricus* and *Lactobacillus acidophilus*, are potentially effective for combating obesity and overweight, particularly when multiple strains are used instead of a single strain. The majority of the studies indicated in the review however were done in Western^(15)^ and Southeast Asian populations^(16–19)^. In fact, there is a scarcity of evidence with respect to Arab ethnic groups where cardiometabolic disorders are common. Hence, the present study aimed to examine the potential anti-obesity effects of multi-strain probiotic supplementation in Arab individuals suffering from overweight or obesity. The use of multi-strain probiotics that contain *Bifidobacterium* strains is of particular interest as it has been shown to affect visceral fat distribution, at least in animal models^(20)^. Consequently, the present study aims to determine whether such strains in combination with others will elicit the same favourable effects in overweight and obese humans.

## Methods

### Study design

This study is a 12-week, single centre, double-blind, randomised, placebo-controlled trial conducted at the occupational health clinics of King Saud University Medical City, Riyadh, Saudi Arabia.

### Subjects

Recruitment was done for students and employees of King Saud University, who received a study invitation and registration link via e-mail. Power calculation was done using G-Power software (version 3·0·10) following the probiotic intervention effect reported by Gomes *et al.*, using waist circumference (WC) as a primary outcome. The obtained power calculation required *n* 17 participants/group (95 % power, 5 % type 1 error) to detect a difference in WC^(21)^. Enrolment of participants was substantially increased taking into consideration large dropouts.

**Fig. 1:**
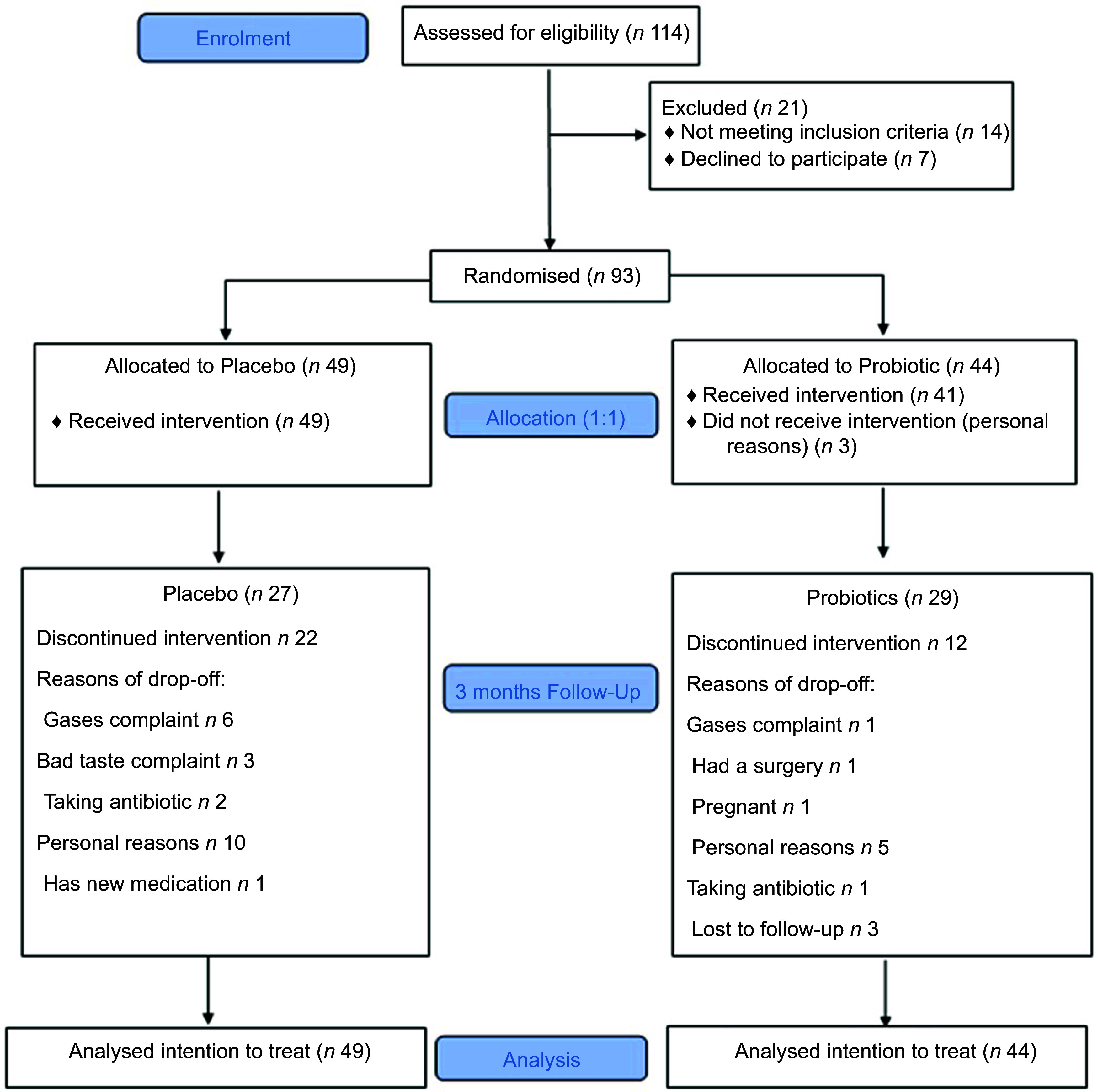
Flowchart of the study subjects describing their participation and allocation

The inclusion criteria included adult Saudi healthy volunteer adults, males and females who were overweight (BMI, BMI 25–29·9 kg/m^2^), obese (BMI 30–34·9 kg/m^2^) and/or abdominally obese (defined as having WC >88 cm in females and >102 cm in males), aged 19–40 years, with relatively stable body weight in the last 3 months before the trial. Subjects who suffer from diseases that may affect weight, such as immune system diseases, thyroid disorders, diabetes type 1, diabetes type 2, any type of cancer, neurological disorders, psychiatric disorders and kidney failure, were excluded. Additionally, lactating or pregnant women, those who had gastrointestinal conditions or surgery, on hormone replacement therapy and on antibiotics or probiotic/prebiotic supplements 2 weeks before the trial, were excluded. Eligible subjects were asked to sign consent prior to enrolment and were blinded to the allocation of treatment. Figure 1 shows the study flowchart.

### Blinding

Allocation of treatment was blinded for all participants in this study, including investigators, research staff or subjects. The study product, HEXBIO, MCP^®^ BCMC^®^ strains, was supplied by B-Crobes Laboratory Sdn. Bhd (Ipoh, Malaysia) in sachets packed and coded as numbers. Unblinding was done at the end of the intervention proper, with a formal request letter sent to the company to unblind the study product.

### Randomisation

Subjects were allocated in blocks based on their sex, age and BMI. From those blocks, a list of pairs was generated and coded as ‘1’ or ‘2’. The list was sent to the inpatient pharmacy for allocation. The randomisation scheme was computer-generated using MS Excel, in which one subject was assigned either code ‘1’ or ‘2’.

### Treatment

A hypoenergetic diet was applied to subjects in both groups, and they were asked to stabilise their physical activity during the entire intervention period, which started 1 week after the first visit and extended until the 12th week. Each subject received three boxes containing either probiotics or placebo, each weighed 3 g and were supplied by HEXBIO® B-Crobes Laboratory Sdn Bhd, Ipoh, Malaysia, which were indistinguishable in terms of colour, weight and shape. The probiotic sachet comprises a granular powder consisting of six strains of microorganisms (30 × 10^9^ CFU). Both the placebo and the probiotic contained the same excipients, with the key difference being that the placebo did not include the live bacteria present in the probiotic. Two sachets should be ingested daily by dissolving their contents in approximately 50 ml of room-temperature water: the first sachet used 10 min prior to the first meal, and the second sachet consumed 10 m prior to the last meal. To promote compliance among subjects with the study instructions, regular contact was used via WhatsApp or phone calls, with weekly communication during the first month and then monthly for the remainder of the intervention period.

### Energy-restricted diet and intake

Energy requirements were assessed using the Saudi Ministry of Health https://www.moh.gov.sa/en/HealthAwareness/MedicalTools/Pages/CalorieCalculate.aspx, taking into consideration sex, age and the type of daily physical activity for each of the study subjects. Given that the study’s target population consisted of overweight and obese adults, a hypoenergetic diet was prescribed to all participants, with a reduction of 300–500 calories tailored to each participant’s needs. Each participant received a personalised calorie calculation guide and a healthy eating guideline developed by the Saudi Ministry of Health. According to the Food Calorie Calculator for Weight Loss on the Saudi Ministry of Health, the recommended distribution of energy is as follows: 45–60 % carbohydrates, 20 % protein and 15–35 % fat. To ensure adherence to the study protocols, regular follow-up was maintained through WhatsApp or phone calls, with weekly check-ins during the first month and monthly follow-ups for the remainder of the intervention period. Dietary intake was evaluated using the 24-h dietary recall questionnaire during the 3 d of the week with 1 d during the weekend. All macronutrients were analysed using a validated food processor nutrition analysis software (ESHA Research).

### Physical activity and anthropometric measurements

The physical activity was assessed at baseline and follow-up using the International Physical Activity Questionnaire (IPAQ), short form, self-administered format^(22)^. Anthropometrics included weight, WC, hip circumferences (HC) and height; they were assessed with the subject wearing lightweight clothing. Weight was measured before breakfast, using a calibrated column scale to the nearest 0·1 kg. WC and HC were measured to the nearest 0·5 cm using a standard tape measure. Height was taken using stadiometer to the nearest 0·5 cm. Based on anthropometric data, the body adiposity markers were estimated depending on the following equations:

- BMI = weight (kg)/height^2^ (m).
- Conicity Index (CI) = 
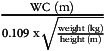
 ^(23)^.

### Biochemical analyses

All analyses were done at the Chair for Biomarkers of Chronic Diseases, King Saud University, using the colorimetric method. Fasting blood samples were taken twice, at baseline and at the end of the intervention. The Konelab routine analyzer (Konelab) was used for routine analysis of fasting blood glucose (FBG), lipid profiles including total cholesterol (TC), HDL-cholesterol and TAG. LDL-cholesterol was calculated using the Friedewald equation, according to Whelton *et al.* ^(24)^. The D-10 device (BIO-RAD) was used to determine HbA1c levels.

### Data analysis

Data were analysed SPSS version 23·0 (IBM SPSS). Categorical data were presented as frequencies (N) and percentages (%). Independent *t* test and Mann–Whitney *U* tests were used to compare baseline differences between groups. Repeated measure ANOVA was used to assess main and interaction effects. Bonferroni corrections were applied to adjust for multiplicity. Intent-to-treat analysis was done, and the last observation carried forward method was applied in case of missing values in all variables. Per-protocol analysis was applied only to the primary outcome (WC). A *P*-value <0·05 was considered statistically significant.

## Results

This section may be divided by subheadings. It should provide a concise and precise description of the experimental results and their interpretation, as well as the experimental conclusions that can be drawn.

### Baseline characteristics of subjects

Baseline characteristics of subjects are summarised in Table 1. No significant differences between groups were observed in all parameters. Majority of the subjects were females (sixty-five females and twenty-eight males). The number of dropouts was *n* 34 (36 %), and this created an uneven allocation in the final analysis (Fig. 1). No serious adverse effects or symptoms were reported with the study product; however, there was a presence of the normal expected temporary side effects, including bloating, diarrhoea and colic sometimes.

**Table 1:** Baseline characteristics of subjects

Parameters	Probiotic		Placebo		*P*-value
	Mean or median	sd or (Quartile 1–Quartile 3)	Mean or median	sd or (Quartile 1–Quartile 3)	
N	44		49		
Age (years)	30·2	5·7	30·4	6·2	0·91
Sex (M/F)	16/28		12/37		0·21
BMI (kg/m^2^)	30·8	2·8	30·8	2·8	0·95
Weight (kg)	82·9	11·7	80·9	10·8	0·40
WC (cm)	92·2	12·5	90·3	10·6	0·42
HC (cm)	114·4	7·9	113·8	6·5	0·71
WHR	0·8	0·1	0·8	0·1	0·46
CI	1·2	0·1	1·2	0·1	0·46
Systolic BP (mmHg)	122·8	11·6	117·9	12·8	0·06
Diastolic BP (mmHg)	72·4	7·8	72·3	9·2	0·94
Energy (kcal)	1656·2	1227–1983	1414·1	1166–1762	0·41
Protein (g)	65·6	52·6–81·5	60·7	44·6–72·9	0·10
Carbohydrate (g)	194·1	151·1–251·7	174·8	150·7–225·1	0·42
Fibre (g)	13·4	9·6–15·7	12·6	9·1–17·5	0·68
Fat (g)	67·0	42·2–81·2	58·8	46·0–77·3	0·82
Physical activities (MET)	936·0	438–1710	693·0	339–1098	0·22
Sitting time (hours)	5·0	4·0–6·0	5·5	4·0–8·0	0·55
HbA1c (%)	5·4	0·4	5·4	0·7	0·69
FBG (mmol/l)	5·1	4·2–6·8	5·1	4·0–6·3	0·68
TC (mmol/l)	5·9	4·1–7·1	5·2	4·2–8·1	0·61
HDL-cholesterol (mmol/l)	1·3	0·6–2·1	1·2	0·6–1·8	0·75
LDL-cholesterol (mmol/l)	3·3	2·2–4·6	3·2	2·4–6·0	0·66
TAG (mmol/l)	1·3	0·8–1·8	1·4	1·0–2·0	0·40

**Note**: Data presented as mean ± sd for normal and median (Quartile 1–Quartile 3) for non-normal variables. WC, waist circumference; HC, hip circumference; WHR, waist-hip ratio; CI, Conicity Index; BP, blood pressure; HbA1c, glycated Hb; FBG, fasting blood glucose; TC, total cholesterol; ST, sitting time; PA, physical activities; MET, metabolic equivalent tasks. *P*-values obtained from independent *t* test and Mann–Whitney *U* test for normal and non-normal variables, respectively; *P* < 0.05 considered significant.

### Primary outcome and anthropometric results

**Fig. 2:**
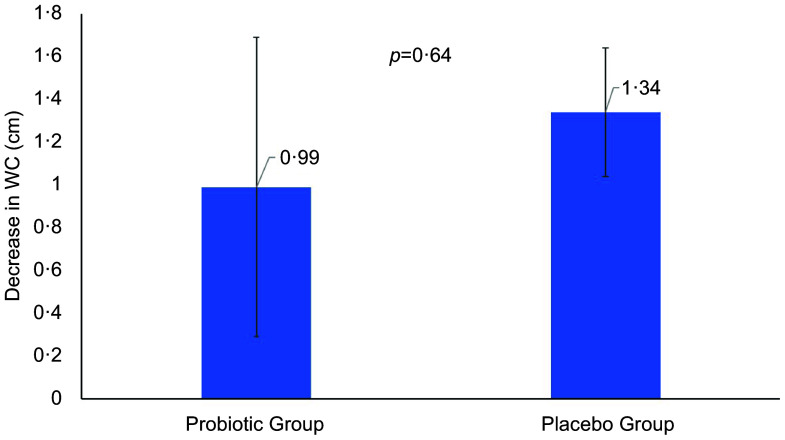
Mean changes in waist circumference overtime in both groups

**Fig. 3:**
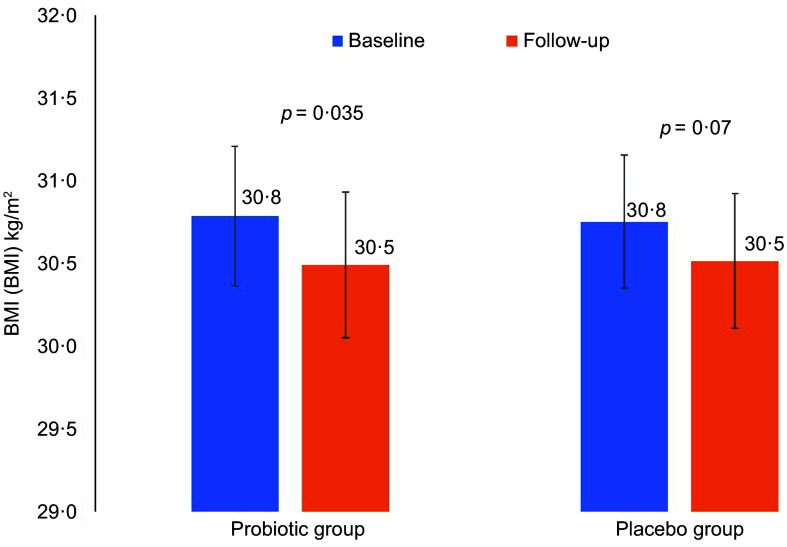
Mean changes in BMI overtime in both groups

**Fig. 4:**
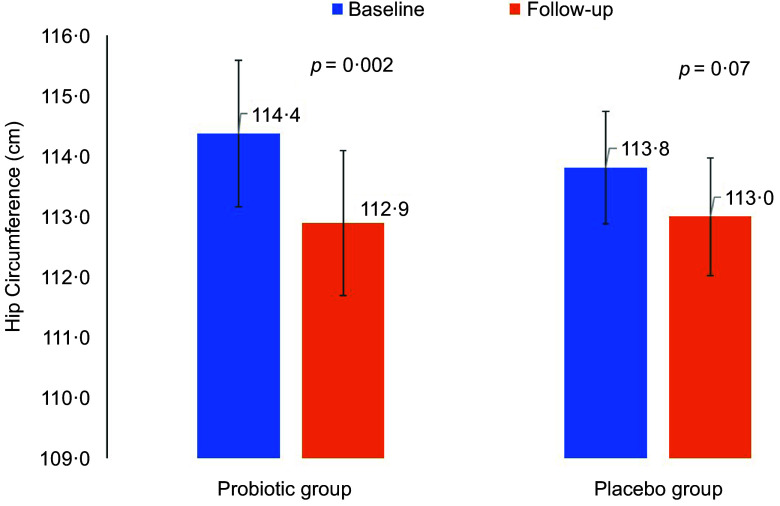
Mean changes in hip circumference overtime in both groups

Differences in anthropometrics using intent-to-treat analysis were summarised in Table 2. The primary outcome was the difference in WC between groups. Between-group comparisons showed no significant differences in primary outcome and other indices. Per-protocol analysis of the primary outcome revealed no clinically significant difference in WC as indicated by insignificant main (*P* = 0·23) and interaction effects (*P* = 0·41) (not included in the table). Mean changes in WC were also NS (Fig. 2). However, many body measurements were significant at 12 weeks favouring the probiotic group, including WC, which modestly decreased over time (*P* = 0·07), and body weight (–0·9 kg, *P* = 0·02), with no significant change noticed in the placebo group. A significant reduction in BMI (*P* = 0·04) and HC (*P* = 0·002) was also observed in the probiotic group post-intervention (Figs. 3 and 4). Significant changes were also noticed in placebo group, including lower WC (*P* = 0·01) and CI (*P* = 0·03).

**Table 2:** Baseline and post-intervention changes in anthropometrics

Parameters	Probiotics (*n* 44)					Placebo (*n* 49)					Group effect	Group* time
	Baseline		Follow-up		*P*-value	Baseline		Follow-up		*P*-value		
	Mean or Median	sd or (Quartile 1–Quartile 3)	Mean or Median	sd or (Quartile 1–Quartile 3)		Mean or median	sd or (Quartile 1–Quartile 3)	Mean or median	sd or (Quartile 1–Quartile 3)			
BMI (kg/m^2^)	30·8	2·8	30·5	2·9	0·04	30·8	2·8	30·5	2·8	0·07	0·99	0·76
Weight (kg)	82·9	11·7	82·0	11·9	0·02	80·9	10·8	80·3	11·3	0·11	0·44	0·55
WC (cm)	92·2	12·5	91·2	13·3	0·07	90·3	10·6	88·9	11·1	0·01	0·39	0·64
HC (cm)	114·4	7·9	112·9	7·8	0·002	113·8	6·5	113·0	6·8	0·07	0·88	0·30
WHR	0·8	0·1	0·8	0·1	0·87	0·8	0·1	0·8	0·1	0·24	0·37	0·35
CI	1·2	0·1	1·2	0·1	0·39	1·2	0·1	1·2	0·1	0·03	0·37	0·39
Systolic BP (mmHg)	122·8	11·6	122·8	11·4	0·97	117·9	12·8	116·0	12·0	0·17	0·02	0·89
Diastolic BP (mmHg)	72·4	7·8	72·4	8·6	1·00	72·3	9·2	71·2	7·9	0·22	0·68	0·40

**Note**: Data presented as mean ± sd for normal and median (Quartile 1–Quartile 3) for non-normal variables; significant at *P* < 0.05. WC, waist circumference; HC, hip circumference; WHR, waist-hip ratio; CI, Conicity Index; BP, blood pressure. *P*-values obtained from repeated measures ANOVA; *P* < 0.05 considered significant.

A total of sixty-three participants were obese (thirty probiotics, thirty-three placebo), and thirty (fourteen probiotic, sixteen placebo) were overweight. Six (9·5 %) participants became overweight from obese (three (10·0 %) probiotic, three (9·1 %) placebo, *P* = 0·90). Four (13·3 %) participants became normal from overweight (two (14·3 %) probiotic, two (12·5 %) placebo, *P* = 0·89), whereas two became obese from overweight (two probiotic). A total of forty participants (nineteen probiotic, twenty-one placebo) had higher WC (WC > 102 for men and > 88 for women). Eight (20·0 %) participants with higher WC became normal (WC ≤ 102 for men and ≤ 88 for women) (four (21·1 %) probiotic, four (19·0) placebo, *P* = 0·87). Only one participant increased their WC to abnormal level (WC > 102 for men and > 88 for women) who belonged to probiotic group. A total of eighteen participants (eleven probiotic, seven placebo) were above the threshold of abdominal obesity (in men as a waist-to-hip ratio of at least 0·90; for women, it’s a ratio of 0·85 or more). Out of eleven, three (27·3 %) became normal in probiotic group, while no one in the placebo group reduced their WHR to normal level (*P* = 0·130). The suggested CI cut-off points to diagnose obesity and metabolic abnormalities are 1·200 for males and 1·180 for females. A total of thirty-seven participants (eighteen probiotic, nineteen placebo) were above the threshold. Four (10·8 %) participants became normal (two (11·1 %) probiotic, two (10·5 %) placebo, *P* = 0·95).

#### Changes in dietary intake and physical activities post-intervention

Changes in dietary intake post-intervention are shown in Table 3. Between-group analysis showed no differences, but significant group*time interaction showed that fat intake decreased significantly in favour of the probiotic group (*P* = 0·02), while within-group analysis showed that there was a significant reduction in the energy (*P* = 0·002), protein (*P* = 0·007) and fat intake (*P* = 0·001) post-intervention, but only in the probiotic group. On the other hand, intake of carbohydrate and fibre was significantly reduced in the placebo group (*P*-values 0·04 and 0·01, respectively). No significant differences were observed in physical activity and sitting hours post-intervention (Table 3).

**Table 3:** Baseline and post-intervention changes in dietary intake and physical activity

Parameters	Probiotic group (*n* 44)					Placebo group (*n* 49)					Group effect	Group* time
	Baseline		Follow-up		*P*-value	Baseline		Follow-up		*P*-value		
	Median	Quartile 1–Quartile 3	Median	Quartile 1–Quartile 3		Median	Quartile 1–Quartile 3	Median	Quartile 1–Quartile 3			
Calories	1656	1227–1983	1269	1060–1654	0·002	1414	1165–1760	1351	1152–1720	0·09	0·73	0·38
Protein (g/d)	65·6	52·6–81·5	59·1	44·7–69·5	0·007	60·7	44·6–72·9	56·0	45·7–67·5	0·53	0·24	0·17
CHO (g/d)	194·1	151–252	178·8	132–224	0·06	174·8	151–225	164·6	142–201	0·04	0·35	0·77
Fibres (g/d)	13·4	9·6–15·7	11·0	8·4–15·1	0·07	12·6	9·1–17·5	10·8	8·6–14·8	0·01	0·78	0·52
Fat (g/d)	67·0	42·2–81·2	47·6	36·0–66·8	0·001	58·8	46·0–77·3	55·2	43·9–71·1	0·41	0·42	0·02
PA MET	936·0	438–1710	826·5	495–1638	0·80	693·0	339–1098	678·0	396–1098	0·49	0·20	0·69
Sit (hours)	5·0	4·0–6·0	5·0	4·0–7·0	0·89	5·5	4·0–8·0	6·0	4·0–7·0	0·73	0·60	0·73

**Note**: Data presented as median (Quartile 1–Quartile 3); significant at *P* < 0.05. CHO, carbohydrates; ST, sitting time; PA, physical activities; MET, metabolic equivalent tasks. *P*-values obtained from repeated measures ANOVA; *P* < 0.05 considered significant.

#### Changes in biochemical parameters post-intervention

Table 4 shows changes in the glycaemic and lipid profiles during the intervention. The between-group analysis was NS; however, within-group analysis showed that there was a significant reduction in HbA1c in both probiotic and placebo groups *(P* = 0·001*)*, while the FBG reduced only in the probiotic group *(P* = 0·002*)*. The lipid profile results changed by the end of the intervention; both TC and LDL-cholesterol reduced within the probiotic group, but this reduction was significant in LDL-cholesterol only (*P* = 0·02); however, TAG increased within both the probiotic and placebo groups (*P*-values 0·001 and 0·01, respectively) (Table 4).

**Table 4:** Baseline and post-intervention changes in glycaemic and lipid profiles

Parameters	Probiotic group (*n* 44)					Placebo group (*n* 49)					Group effect	Group*time
	Baseline		Follow-up		*P*-value	Baseline		Follow-up		*P*-value		
	Mean or median	sd or interquartile range	Mean or median	sd or interquartile range		Mean or median	sd or interquartile range	Mean or median	sd or interquartile range			
HbA1c (%)	5·4	0·4	5·0	0·6	<0·001	5·4	0·7	5·2	0·8	<0·001	0·42	0·26
FBG (mmol/l)	5·1	4·2–6·8	4·4	3·9–5·4	0·002	5·1	4·0–6·3	4·8	4·3–5·8	0·36	0·60	0·14
TC (mmol/l)	5·9	4·1–7·1	4·9	4·1–6·1	0·17	5·2	4·2–8·1	5·2	4·7–6·5	0·39	0·36	0·80
HDL-cholesterol (mmol/l)	1·3	0·6–2·1	1·1	0·7–1·6	0·27	1·2	0·6–1·8	1·1	0·8–1·6	0·61	0·96	0·72
LDL-cholesterol (mmol/l)	3·3	2·2–4·6	2·6	1·8–3·8	0·02	3·2	2·4–6·0	3·2	2·2–4·0	0·22	0·23	0·52
TAG (mmol/l)	1·3	0·8–1·8	2·2	1·3–3·2	0·001	1·4	1·0–2·0	2·0	1·2–2·9	0·01	0·95	0·31

**Note**: Data presented as mean ± sd for normal variables, while non-normal variables are presented as median (interquartile range); significant at *P* < 0.05. HbA1c, glycated Hb; FBG, fasting blood glucose; TC, total cholesterol. *P*-values obtained from repeated measures ANOVA; *P* < 0.05 considered significant.

## Discussion

The main findings of the present study demonstrated that although multi-strain probiotic supplementation for 12 weeks among Arab adults with overweight or obesity had statistically significant effects on many anthropometric measurements, including body weight, BMI and HC, these effects were not clinically meaningful when compared with placebo, with the exception of fat intake. These findings are in opposition with Michael *et al.*, who used a multi-strain of *lactobacilli* and *bifidobacteria* (5 × 10^10^ CFU). They found a significant weight reduction favouring the probiotic group after 6 months of treatment (–1·30 kg, *P* = 0·0001). Also, the reduction was significant in BMI (−1·5 %, *P* < 0·0001), WC (−0·9 %, *P* < 0·0001) and WHR (−1·2 %, *P* < 0·0001). Interestingly, the reduction was greater in individuals who were overweight (−1·9 %, −1·5 kg) than individuals with obesity (−1·2 %, −1·06 kg)^(25)^. Additionally, in a 3-week randomised controlled trial, consumption of probiotic-fortified cheese led to a number of advantageous changes in health indicators among individuals with overweight or obesity; the reduction was larger in the probiotic group than the control group in body weight (−5·7 *v*. –4·4 kg, *P* = 0·08) and BMI (−2 *v*. –1·6 kg/m^2^, *P* = 0·03), suggesting probiotic positive effects on metabolic disorders^(26)^. Likewise, greater differences in body measurements were noticed for subjects who took the multi-strain probiotic (1 × 10^9^ CFU) combined with a diet than the group who only applied a diet. The decrease was in WC (*P* = 0·03), WHR (*P* = 0·02) and CI (*P* = 0·03)^(21)^. Furthermore, the administration of *Lactobacillus gasseri* SBT2055 for healthy adults revealed a significant decrease in the visceral, subcutaneous, total fat areas, body weight, BMI, WC, HC and WHR within the probiotic group and between-group comparisons at baseline and week 12, demonstrating that the inhibition of lipid absorption is a possible mechanism underlying the observed effects^(27)^. In another randomised controlled trial, taking the probiotic *Lactiplantibacillus plantarum* IMC 510^®^ (1·5 × 10^10^ CFU) for 3 months led to a significant drop in body weight (*P* = 0·03), BMI (*P* = 0·03), WC (*P* = 0·04) and WHR (*P* = 0·04)^(28)^. The favourable effects observed within the probiotic group in the present study may be attributed to probiotic supplementation, considering that a hypoenergetic diet was applied to both the probiotic and placebo groups. The lack of clinically significant effect as compared with previous findings can be attributed to ethnic groups, as different populations may exhibit varying lag time in biological response to probiotic supplementation. According to Gupta and colleagues, geographical variations in microbiome structure can significantly affect how a population responds to microbiome-based therapeutics, including probiotics^(29)^. Additionally, based on the existing understanding of the effectiveness of probiotic supplementation in mitigating overweight and obesity, current systematic review emphasised that probiotic genus, strain, dosage, duration of supplementation and delivery matrix were known as significant factors on the anti-obesity effects of probiotics^(30)^.

Similar to the present findings, one study assessed the effects of probiotic supplementation on weight reduction in healthy, young adult females using a 6-week supplementation with *Bifidobacterium lactis BS01* and *Lactobacillus acidophilus LA0* (2 × 10^9^ CFU) without involving dietary restrictions. The findings showed that the BMI decreased higher in the supplemented group after treatment (by 4·1 % compared with 0·81 % in the placebo group); however, the differences were NS. WC was elevated by 0·67 % in the supplemented group and reduced by 1·33 % in the placebo group. Changes in either group were not statistically significant. Similarly, WHR increased by 1·195 % in the supplemented group and decreased by 1·36 % in the placebo group. The effect sizes were all modest and insignificant^(31)^. Among treatment naïve subjects with type 2 diabetes, a significant improvement in WHR (*P* = 0·02) was observed favouring the probiotics group when compared with the placebo group, and no differences were noted in weight or BMI. However, within the group, this significant alteration was absent in weight, BMI and WHR following the administration of multi-strain probiotics over a period of 3 months^(32)^. Moreover, Zarrati and colleagues used three different groups for the intervention: probiotic yogurt enriched in multi-strain *Lactobacillus* and *Bifidobacterium* with a low caloric diet (PLCD), probiotic yogurt without a low caloric diet and regular yogurt with a low caloric diet (RLCD). The results revealed that the RLCD group had a greater reduction in body weight, BMI and HC (–24·87, –21·9 and –23·18, respectively) compared with the PLCD group (–24·23, –21·55 and 21·84, respectively) after the intervention. However, all changes were NS. In contrast, among all assessed variables, only WC changes were larger in the PLCD group compared with the RLCD group (–2·78 and –2·3, respectively), although this difference was still statistically insignificant (*P* = 0·7)^(33)^. Similarly, Omar and others reported no significant differences in body weight or fat mass at the conclusion of the study; body weight fluctuations were less than 5 %^(34)^.

Regarding dietary intake, the probiotic group showed a significant reduction in calories compared with the baseline (–387·3 kcal/d, *P* = 0·002), but this reduction was not statistically significant when compared with the placebo group (–63 kcal/d, *P* = 0·09). This reduction is suggested to be primarily due to the decrease in fat intake (–29 %, *P* = 0·001) in the probiotic group, while the placebo group exhibited a 6 % reduction in fat intake (*P* = 0·41) compared with the baseline. Consuming excessive dietary fat not only elevates the body’s exposure to potentially pro-inflammatory free fatty acids and their derivatives but also suppresses the expression of tight junction proteins, such as zonulin and occludin, thereby increasing intestinal permeability^(35)^. This promotes the absorption of endotoxins leading to metabolic endotoxemia. Therefore, Saudi clinical practice guideline emphasises on adopting a low-calorie diet with a targeted reduction in fat intake to less than 30 %^(36)^. Likewise, Mahadzir *et al.* reported that after a 4-week period of MCP (3·0 × 10^10^ CFU) consumption, the same product of this study, the subjects in the probiotic group exhibited a significant decrease (*P* = 0·04) in their energy consumption, roughly 300 kcal/d, in comparison with their initial intake levels^(37)^. Additionally, the reduction in food intake was also noticed in the Canadian study, but it was non-significant; however, the energy intake seemed to be consistently lower in the women probiotic group when compared with women in the placebo group^(38)^. In contrast with current results, the findings of a study carried out in Japan, there was no significant difference in the intake of energy or main nutrients in the groups at any of the three different time points (week 4, week 8 and week 12)^(27)^. Hence, the observed reductions in body measurements cannot be solely attributable to changes in calorie consumption; instead, they may be attributable to the impact of synbiotics on the GM of individuals, hence inducing alterations in energy metabolism and perhaps facilitating weight reduction irrespective of calorie limitation^(39)^.

The mechanisms behind the reduction in anthropometrics and food intake are intertwined. The alteration of host energy homeostasis is one mechanism to reduce weight^(8)^, which includes the harvesting, storing and expenditure of energy obtained from the diet. For example, a 20 % increase in *Firmicutes* and a 20 % decrease in *Bacteroidetes* were associated with an additional energy harvest of 150 kcal/d^(5)^. Also, SCFA, which are by-products of microbial metabolism, may help regulate host homeostasis in different ways. For example, SCFA affect the production of serotonin (5-HT), which prolongs satiety and reduces food intake^(40)^. SCFA can also induce satiety in other ways; for example, acetate and propionate stimulate leptin secretion^(41)^; butyrate releases glucagon-like peptide-1^(42)^. Additionally, through the postprandial phase, both glucagon-like peptide-1 and peptide YY are also produced in the intestine under the effect of SCFA^(43,44)^. Also, Backhed and his colleagues said that the change in body composition caused by probiotics could be the result of fasting-induced adipose factor suppression in the gut, which would change the production of SCFA^(45)^.

Similar to the present study findings, applying a hypoenergetic diet and probiotic cheeses lowered the values of FBG by 18 % in the treatment and control groups; yet only the control group achieved statistical significance^(26)^. Administration of a multi-strain probiotic supplement over a period of 6 months also resulted in a significant reduction in circulating FBG (38 %) as compared with the baseline among treatment naïve subjects with T2DM; however, there were no statistically significant differences in FBG between the placebo and probiotic groups at both the 3-month and 6-month time points^(46)^. In a 3-month intervention using a multi-strain probiotic for athletes, both the treatment group and the control group of female participants saw a drop in their FBG and HbA1c levels. In addition, a beneficial decrease in FBG concentration was observed in the male participants who received the probiotic intervention, whereas there was an increase in the male participants who received the placebo^(47)^.

In terms of lipid profile, no significant changes between groups were observed in the present study. Other findings confirm that multispecies probiotics have a positive effect on the lipid profile of postmenopausal women with obesity^(48)^. Another randomised controlled trial, which lasted for 3 weeks, evaluated the effect of a hypoenergetic diet with 50 g/d of full-fat probiotic cheese containing *L. plantarum* TENSIA on the lipid profiles. The combination of diet and probiotics reduced the values of TC and LDL-cholesterol significantly in the treatment group and control groups, while HDL-cholesterol and TAG were significantly reduced in the treatment group only^(26)^. In the same context, a meta-analysis finding found that probiotic yogurt significantly lowers TC and LDL-cholesterol levels in subjects with mild to moderate hypercholesterolaemia, specially, studies lasting more than 4 weeks, but there was no significant effect on HDL-cholesterol or TAG levels^(12)^. Additionally, Sabico *et al*. reported the consumption of multi-strain probiotics having significant benefits in terms of reduced TAG (48 %), TC (19 %) and the total/HDL-cholesterol ratio (19 %) in the probiotic group^(46)^. In contrast, Michael *et al.* noticed that the TC, HDL-cholesterol and TAG levels remained unchanged through 6 months for the study population; moreover, LDL-cholesterol levels increased 2·7 % from baseline in both the probiotic group (0·087 mmol/l, *P* = 0·07) and the placebo group (0·088 mmol/l, *P* = 0·06)^(25)^. Similarly, Kadooka *et al.* also found no significant changes in both lipid metabolism-related parameters, such as TC, LDL-cholesterol and HDL-cholesterol, and physiological parameters, such as blood test, urine test, blood pressure or pulse rate^(27)^.

Several hypotheses have been suggested on the mechanisms by which probiotics can reduce cholesterol levels, based mostly on *in vitro* research, for instance, bile salt hydrolase activity, binding of cholesterol to the probiotic cellular surface and production of SCFA^(49)^. Bile salt hydrolase, which is expressed in probiotic strains, deconjugates bile salt to become less efficiently reabsorbed than conjugated bile acids, leading to the excretion of significant amounts of free bile acids in human faeces; thus, more cholesterol is needed to replace excreted bile salt, which ultimately reduces TC in the blood^(50)^.

The study’s limitations include its duration of 12 weeks. Despite observing significant changes within the probiotic group, the absence of clinically significant differences between groups suggests that extending the treatment period might be necessary to achieve noticeable clinical outcomes. Michael *et al.* (2020) demonstrated that a meaningful impact on individuals with obesity typically requires at least 6 months of supplementation with a multi-strain probiotic^(25)^. Nonetheless, this study holds significance as the first of its kind to investigate the anti-obesity effects of multi-strain probiotics in a homogeneous ethnic Arab Saudi adult population. Moreover, it maintains merit due to its implementation of adequate statistical power and rigorous blinding procedures. Furthermore, the intervention protocol allowed for an assessment of probiotics in conjunction with the benefits of a hypoenergetic diet intervention.

## Conclusions

In conclusion, a 12-week supplementation of multi-strain probiotics among overweight or obese Saudi adults showed beneficial effects on anthropometric indices, FBG and LDL-cholesterol compared with baseline, with no such improvements observed in the placebo group. However, these changes did not reach clinical significance with the exception of dietary fat intake in favour of the probiotics group. Future research should consider longer trial durations to verify whether alterations in GM composition would lead to clinically meaningful outcomes following extended probiotic intake.

## Data Availability

Data that have been collected or analysed for the current study are accessible from the corresponding author upon reasonable request.
